# Importance of palm's heart for pregnant women

**DOI:** 10.1017/jns.2022.112

**Published:** 2023-01-12

**Authors:** Efrem Negash Kushi, Tefera Belachew, Dessalegn Tamiru

**Affiliations:** 1 College of Health and Medical Science, Mettu University, Mettu, Ethiopia; 2 Departments of Nutrition and Dietetics, Jimma University, Jimma, Ethiopia

**Keywords:** Nutritional value, Palm heart, Pregnant women, West Ethiopia, AOAC, Association of Official Analytical Chemists’ methods, kcal, kilocalories, RDA, Recommended Dietary Allowance, WHO, World Health Organization

## Abstract

The consumption of locally nutrient-rich edible plants in rural areas can be used to satisfy the dietary diversity of pregnant women. Date palm is one of the wild edible plants in different parts of the world. Studies on wild edible plants in Ethiopia cover only about 5 % of the country's districts. Furthermore, the nutrient composition of the palm heart of *Phoenix reclinata* is not yet investigated as it is commonly consumed by indigenous people in western Ethiopia. The utilization of such plants requires strong policy support based on scientific evidence to maintain the nutrition security of pregnant women. Homogeneous samples of 1000 grams (g) of palm hearts were collected randomly. The macronutrient contents were determined using standard methods of the Association of Official Analytical Chemists (AOAC, 2000). The flame Photometric method was used for potassium and sodium determination. The carbohydrate concentration (g/100 g) was 78⋅2. It covers approximately 78⋅5 % of the total daily Recommended Dietary Allowance (RDA). In line with this, the concentrations of minerals such as potassium (K+) and sodium (Na+), per milligram (mg/100 g) of the sample were 1962⋅3 and 7⋅9, respectively. The palm heart of *Phoenix reclinata* has many nutritional values and is important for pregnant women. Its nutrient composition is comparable with different staple foods of the country and can contribute to household food security in rural communities.

## Introduction

Adequate nutrition is critical for pregnant women due to their vulnerability to malnutrition^(1)^. It needs targeted efforts to promote the consumption of local nutrient-rich edible plants^(2)^. Furthermore, maternal nutrition is important for the quality of their lives and their children^(3)^. An adequate diet before pregnancy reduces the chance of preterm and other related complications while the problems of under as well as over-nutrition are common in the female population globally^(3,4)^.

Despite the agricultural potential in rural areas in different parts of the world, food insecurity and poor-quality diets remain a challenge for women^(5)^. Moreover, in rural areas, the local community depends on wild edible plants to satisfy the diversity of their food^(6)^. Wild plants play a role in increasing dietary diversity and as a means of income for women in different parts of the world^(7)^.

Senegal date palm (*Phoenix reclinata*) is one of the wild edible plants in rural communities of western Ethiopia. It is a drought-tolerant plant native to tropical Africa, specifically western Senegal that was distributed from Senegal to Ethiopia^(8,9)^. Palms are important to humans as a food source and 183 genera of the palm family, of which *Phoenix* is one of the genera that consists of 14 species^(10)^. Out of these, *reclinata* is native to tropical Africa, Comoros, Madagascar and Arabian Peninsula^(11)^.

The heart of the palm is an extracted product of the palm which can be prepared in different forms and consumed in salads, soups and other dishes^(12)^. It is an important source of dietary fibre and a good source of vitamins that can be added to any healthy diet^(13)^. Even though the palm's heart is less utilised than edible palm products, it is a major part of the food industry in contrast to most palm products^(14)^.

The nutritional composition and antioxidant capacities of different parts of various palm species such as fruits, hearts and buds were reported in many studies^(12,15–17)^. However, the nutrient composition of the palm heart of *Phoenix reclinata* is not yet investigated. Moreover, studies on wild edible plants of Ethiopia cover only about 5 % of the country's districts when compared with the wild edible plant wealth of the country^(18)^. As a consequence of poverty, and food insecurity, rural population of Ethiopia especially women are highly exposed to malnutrition (undernutrition)^(19)^. Therefore, sustainable harvesting of wild economic species requires strong policy support to maintain nutritional security for local communities of Ethiopia.

## Methods and materials

### Sampling procedure

The samples of palm hearts were collected randomly by experienced people from their natural habitat in rural areas of Asossa and Sherkole districts, West Ethiopia. The tree was cut down and the bark was removed leaving layers of white fibres around the centre core. Then, sampling as many plants as possible and under calm climatic conditions is recommended^(20,21)^. Moreover, samples should be large enough for all intended analyses. In line with this, homogeneous samples of 1000 g are generally sufficient and considered for this study^(22)^. During sampling, samples that had suffered long-term climatic stress, damaged mechanically, infested with disease and contained dead plant tissue were excluded^(23)^.

### Sample preparation

The samples were converted into homogeneous material for various nutritional analyses. Drying and grinding are essential operations since the elemental concentration used for interpretation was based on the dry weight of the sample^(24)^. Therefore, the collected palm heart was washed using running water and stored under freezing temperature. Then, the sample was frozen-dried, and ground to a fine powder in a mixer grinder to 1 millimetre (mm) particle size, stored in polythene bags and transported to the Ethiopian Health and Nutrition Research Institute^(25)^.

### Sample analysis

The proximate composition of the sample for the percentage of moisture content, and macronutrient contents such as crude protein, crude fat, ash contents and crude fibre in the sample was determined using standard methods of the Association of Official Analytical Chemists methods (AOAC, 2000). In line with this, crude fibre was determined using the neutral detergent fibre estimation method in three replicates and the average was considered. But carbohydrate was obtained by calculating the difference (the sum of protein, fat, ash and crude fibre on a dry basis is subtracted from 100)^(26,27)^.

The fat content of the sample was determined using the Soxhlet extraction apparatus (Model Soxtherm Automatic), whereas the ash content was determined by igniting a weighed portion of the dried sample in a muffle furnace (Model Heraeus, Germany) at 550°C and weighing the residue (ash). Finally, the energy content of the sample was calculated using the factors of protein × 4, fat × 9 and digestible carbohydrate × 4^(28)^.

Total nitrogen (crude protein) was determined using the Kjeldahl method. The sample is digested in sulfuric acid using potassium sulfate and copper sulfate (K_2_SO_4_ CuSO_4_/TiO_4_) as a catalyst. Nitrogen is converted into ammonia (NH_3_), then distilled trapped in boric acid, and titrated with hydrochloric acid (HCl). Finally, crude protein was calculated from total nitrogen by multiplying it with 1⋅4007 conversion factor of the total percentage of nitrogen^(29)^. The sample was done in three replicates and the average was taken.

The dry-matter determination is used to correct the sample element concentration to an absolute dry-matter basis. Thus, all nutritional analyses were based on dry matter content. The sample was weighted initially and then inserted in an oven-dried at 65°C for 72 h or 3 d. Likewise, weight was also taken after oven drying. The sample determination for the dry matter was done in three replicates, the average was taken, and was reported to the nearest 0⋅1 %^(30)^.

The amount of potassium and sodium from the sample was determined by the flame Photometric method. Reagents such as: dilute nitric acid (HNO_3_) (1:1 with water), deionised water, sodium stock solution (3⋅05 parts per million (ppm)), potassium stock standard (3⋅15 ppm) and dilute solutions of potassium were used. Furthermore, the flame photometer with 3⋅05 ppm of sodium solution which gives 90 absorbance using a sodium filter was calibrated. Likewise, 3⋅15 ppm of potassium solution which gave 100 absorbances using a potassium filter was also calibrated. Finally, values for the zero-concentration standard were corrected. Then, after wet digestion, the sodium or potassium content was measured using flame photometrically^(31)^.

## Results

The concentration (g/100 g) of dry matter and carbohydrates for the palm heart of *Phoenix reclinata* were found to be 92⋅4 and 78⋅2, respectively. Its crude fat content was 2⋅9 g/100 g. The energy content of the sample was 366 kilocalories per gram (kcal/g) sample. The concentration (g/100 g) of the crude fibre and ash content were 1⋅8 and 10⋅3, respectively. Likewise, the concentration of potassium (K+) per mg/100 g of the sample was 1962⋅3 (Table 1).

**Table 1.** Proximate nutrient composition of palm heart of *Phoenix reclinata*, west Ethiopia, 2022

Parameters	Concentration
Dry matter (g/100 g)	92⋅4
Ash (g/100 g)	10⋅3
Crude fibre (g/100 g)	1⋅8
Crude fat (g/100 g)	2⋅9
Crude protein (g/100g	6⋅8
Sodium (Na) (mg/100 g)	7⋅9
Potassium (K) (mg/100 g)	1962⋅3

## Discussion

The concentration (g/100 g) of carbohydrates for the palm heart of *Phoenix reclinata* was found to be 78⋅2. This is much higher than the heart of date palm from three Saudi varieties: Sukkari (41⋅5), Solleg (44⋅7) and Naboat Saif (50⋅9)^(12,32)^. Its fat content is much higher as compared with Sukkari (1⋅7), Solleg (1⋅8) and Naboat Saif (1⋅6) and also that of okra (7⋅03) and *Moringa oleifera* leaves (38⋅6)^(12,32,33)^. This might be due to environmental factors and soil conditions. This variation may have occurred due to the difference in the climatic conditions of the countries. On the other hand, this result was comparable with the carbohydrate contents of different staple foods of Ethiopia: teff (73), maize (72) and wheat (71)^(34)^. When compared with the World Health Organization (WHO) RDA for carbohydrates, it covers about 78⋅5 % of the total daily RDA for that nutrient which is 130 g. This implies that an intake of palm heart of *Phonex reclinata* is very crucial in reducing malnutrition specifically in rural communities of developing countries like Ethiopia where there is food insecurity.

The fat content of this wild edible plant was comparable with wild vegetables consumed in Bangladesh and the staple food of Ethiopia: teff (2⋅5), wheat (2⋅0) and rice (2⋅2)^(34,35)^. This is also adequate for daily diet. But the crude fat content for this sample was lower when compared with other edible plant leaves of *Moringa oleifera* (17⋅1)^(33)^.

The energy contents of *Phoenix reclinata* are low as compared with fruits of *Phoenix dactylifera* L. (389 kcal/g) and all oat varieties of Ethiopia which ranges from 431 to 439 kcal/g. But this is comparable with that of wild vegetables in Bangladesh which ranges from 326 to 371 kcal/g^(15,35,36)^. The result was comparable with that of the most common staple food of Ethiopia: teff (357 kcal/100 g flour), maize (375 kcal/100 g), sorghum (370 kcal/100 g), wheat (359 kcal/100 g) and rice (357 kcal/100 g)^(34)^. This indicates that the consumption of palm heart of *Phoenix reclinata* as a cabbage plays a great role in maintaining the daily energy needs and preventing energy deficiency problems in rural communities of developing countries^(32)^. However, the energy content per 100 g of the sample was much higher than that of okra (31 kcal/g) and potatoes (310 kcal)^(37)^.

The nutritional composition of *Phoenix* varies from species to species and also differs from the edible plant parts. Accordingly, the concentration of the crude fibre and ash content of the sample was much lower than others^(15,32,35)^. Likewise, this is also lower than that of the staple food of Ethiopia: teff^(34)^. Dietary fibre is important in reducing the formation of free radicals and serves as an important part of a healthy diet^(38)^. In line with this, foods containing considerable amounts of fibre are used in the digestion process (prevents constipation), reduce levels of circulating cholesterol, prevent colon cancer and can also decrease the conversion of starch to simple sugars (prevents diabetics)^(39–41)^. Therefore, the consumption of palm heart of *Phoenix reclinata* as a cabbage plays a very important role in reducing different nutritional-related problems and also alleviation of malnutrition in rural communities of Ethiopia.

The concentration of crude protein in the sample was much lower as compared with others^(35)^. Similarly, as compared with the crude protein content of oat grains cultivated in Ethiopia, that of *Phoenix reclinata* was much lower^(36)^. Out of the total RDA value of protein needed per day, only 3 % is obtained from the palm heart of *Phoenix reclinata*. On the other hand, the crude protein content of the sample was higher than that of fruits of *Phoenix dactylifera* L. and also that of okra and potatoes^(15,32,42)^. There is evidence that indicated that crude protein is used as the enzymatic catalyst which mediates different metabolic process and control cell growth, and differentiation^(43)^. This indicates that natural resources have the potential to play a central role in addressing food insecurity in sub-Saharan Africa like Ethiopia. Thus, the utilisation of nutritive wild edible plants could be cost-effective and a sustainable method of preventing nutritional-related health problems, especially for pregnant women^(44)^.

The palm heart of *Phoenix reclinata* contains a high concentration of potassium and sodium as compared with the chemical composition of different cultivators of fruits of date palm^(45,46)^. Likewise, its potassium content was also higher as compared with those found in okra and eggplant^(32,47)^. These variations could be due to different factors such as a variety of palms, parts of palm used for food, soil type, and fertility, climatic conditions, or stage of plant part taken for sample analysis.

The palm heart of *Phoenix reclinata* had much nutritional importance for pregnant women in reducing the different nutritional deficiencies as a result of their vulnerability. In line with this, wild edible plants are the backbone of dietary diversity, especially for pregnant women in rural communities of developing countries^(48)^. This indicated that indigenous plant foods have great potential in preventing both macro and micronutrient deficiency^(49)^. Similarly, women who consume such food sources had higher intakes of calcium (Ca) and iron (Fe) than those who did not^(50)^. Thus, the consumptions of wild edible plants have much nutritional importance for women which covers about 50 % of their total daily energy intake and other micronutrient needs^(28)^.

## Conclusion

The present study revealed that the palm heart of *Phoenix reclinata* had different nutritional values which are comparable with different staple foods of the country. This prevents different macro and micronutrient deficiencies in pregnant women. Therefore, it has a role in maintaining household food security in rural communities. Promotion and encouragement of its utilisation and detailed scientific studies on its nutritional composition will be needed. Likewise, community awareness of health-promoting components of palm heart of *Phoenix reclinata* in the daily diet of pregnant women, and policy attention are recommended.

## Supplementary material

For supplementary material accompanying this paper visit https://doi.org/10.1017/jns.2022.112. click here to view supplementary material
