# Importance of Palm's heart for pregnant women – CORRIGENDUM

**DOI:** 10.1017/jns.2023.110

**Published:** 2023-12-11

**Authors:** Efrem Negash Kushi, Tefera Belachew, Dessalegn Tamiru

Details: Affiliation to Department of Nutrition and Dietetics, Jimma University, Jimma, Ethiopia, was missing for Efrem Negash Kushi. Corrected author and affiliation displayed below.

Efrem Negash Kushi^1,^^2^*, Tefera Belachew^1^, Dessalegn Tamiru^1^

**1:** Department of Nutrition and Dietetics, Jimma University, Jimma, Ethiopia

**2:** Department of Public Health, Mettu University, Mettu, Ethiopia

